# Supplementary figures and images for: Crumbs and the apical spectrin cytoskeleton regulate R8 cell fate in the Drosophila eye
Source: PLoS Genet. 2021 Jun 7;17(6):e1009146. doi: 10.1371/journal.pgen.1009146 (PMC8211197; doi:10.1371/journal.pgen.1009146)

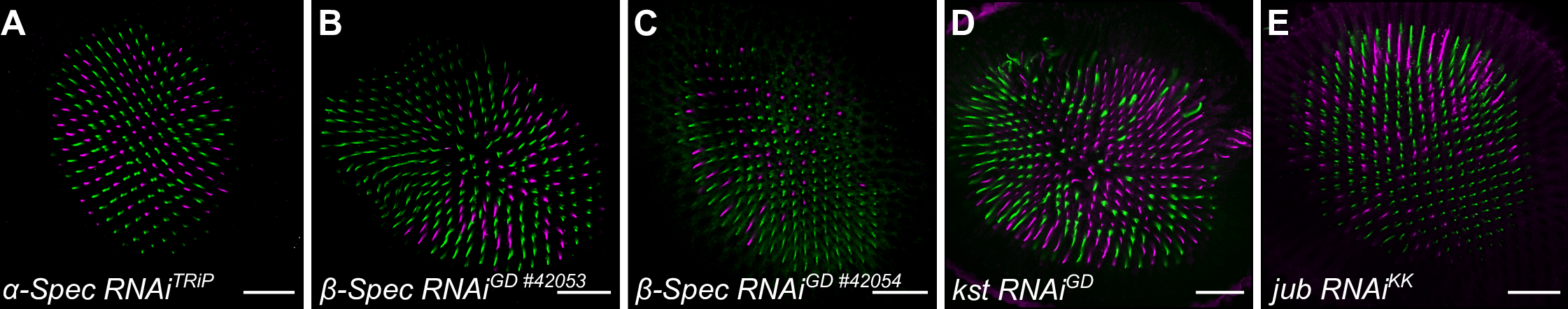

Supplement: S1 Fig — (A-E) Confocal microscope images of adult D. melanogaster retinas stained with anti-Rh5 (magenta) and anti-Rh6 (green) antibodies. The indicated RNAi lines were driven by lGMR-Gal4. Retinas expressed α-Spec RNAiTRiP (A), β-Spec RNAiGD #42053 (B), β-Spec RNAiGD #42054 (C), kst RNAiGD (D) or jub RNAiKK (E). Scale bars are 50μm. (TIF) [file pgen.1009146.s001.tif]

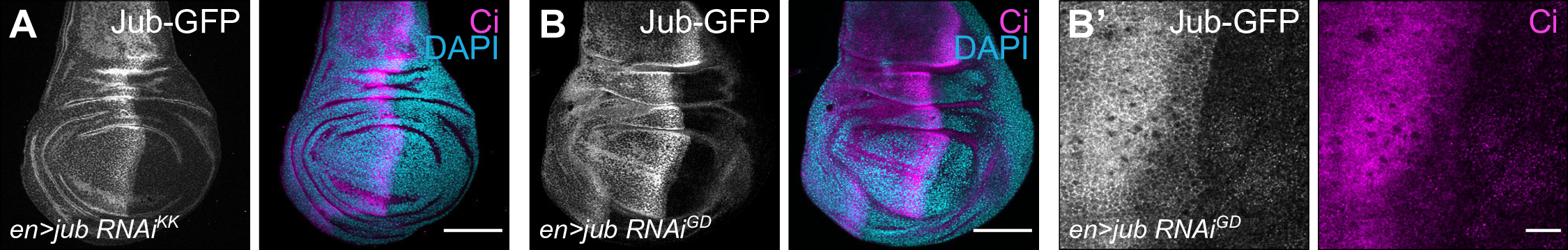

Supplement: S2 Fig — (A-B’) Confocal microscope images of third instar larval D. melanogaster imaginal wing discs from Jub-GFP animals that also expressed one of two jub RNAi lines in the posterior compartment, under control of en-Gal4. Tissues were stained with anti-Ci (magenta) antibody to mark the anterior compartment (left) and DAPI (cyan) to mark nuclei. The genotypes for each tissue are: en>jub RNAiKK; Jub-GFP (A) and en>jub RNAiGD; Jub-GFP (B-B’). Scale bars are 100μm in A and B and 10μm in B’. (TIF) [file pgen.1009146.s002.tif]

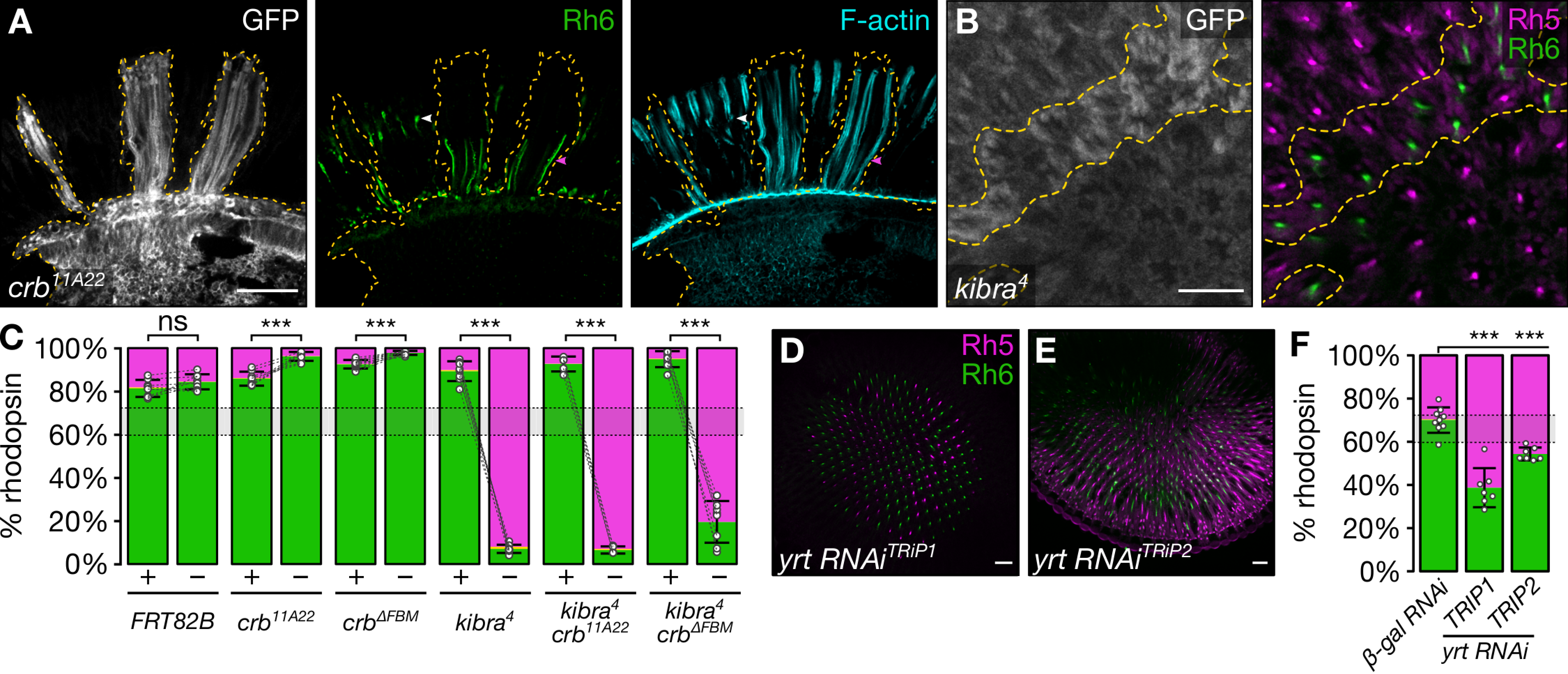

Supplement: S3 Fig — (A) Confocal microscope images of adult D. melanogaster retinas stained with anti-GFP (grey) and anti-Rh6 (green) antibodies and stained with Phalloidin (cyan) to visualise the rhabdomeres. GFP-negative clones were mutant for crb11A22. Arrowheads indicate a wild type ommatidium (magenta) and a mutant ommatidium (white). Scale bar is 50μm. (B) Confocal microscope images of adult D. melanogaster retinas stained with anti-GFP (grey), anti-Rh5 (magenta) and anti-Rh6 (green) antibodies. GFP-negative clones were mutant for kibra4. Scale bar is 50μm. (C) Proportion of R8 cells in wild type (‘+’) or mutant (‘–’) clones that express Rh5 (magenta), Rh6 (green), or both (yellow). Grey lines connect wild type and mutant clones from the same retina. The error bars represent the standard deviation of total % Rh5 (% Rh5 + % Rh5+Rh6). Total % Rh5 was compared with two-sided, unpaired t-tests; ns = not significant, *** = p<0.0001. The shaded grey region between the dotted grey lines indicates wild type Rh5:Rh6 ratio range. FRT82B: n = 8 retinas, 4065 ommatidia; crb11A22: n = 8, 1394; crbΔFBM.HA: n = 10, 3851; kibra4: n = 8, 2776; kibra4 crb11A22: n = 5, 1174; and kibra4 crbΔFBM.HA: n = 8, 2479. (D-E) Confocal microscope images of adult Drosophila retinas stained with anti-Rh5 (magenta) and anti-Rh6 (green) antibodies. Retinas expressed either UAS-yrt RNAiTRiP1 (JF03429) (D) or UAS-yrt RNAiTRiP2 (HMS01532) (E). Scale bars are 20μm. (F) Proportion of R8 cells that express Rh5 (magenta), Rh6 (green), or both (yellow). The error bars represent the standard deviation of total % Rh5 (% Rh5 + % Rh5+Rh6). Total % Rh5 was compared with two-sided, unpaired t-tests; *** = p<0.0001. The shaded grey region between the dotted grey lines indicates wild type Rh5:Rh6 ratio range. β-gal RNAi (Fig 1D): n = 9 retinas, 3976 ommatidia; UAS-yrt RNAiTRiP1 (JF03429): n = 7, 1740; UAS-yrt RNAiTRiP2 (HMS01532): n = 7, 1224. (TIF) [file pgen.1009146.s003.tif]

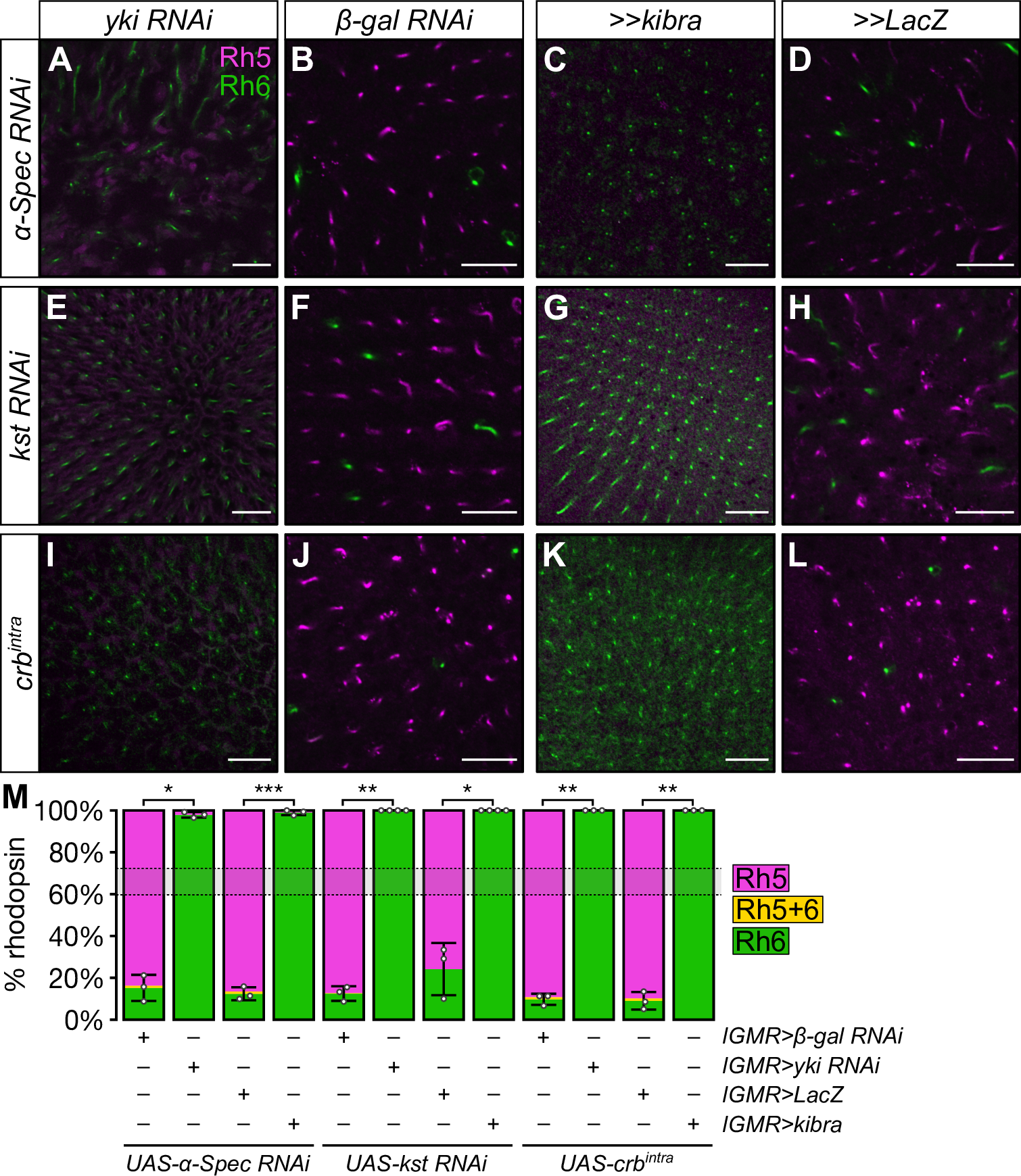

Supplement: S4 Fig — (A-L) Confocal microscope images of adult Drosophila retinas stained with anti-Rh5 (magenta), anti-Rh6 (green) and anti-β-gal (grey; Wts-LacZ) antibodies. Retinas expressed either UAS-α-Spec RNAi (A-D), UAS-kst RNAi (E-H), or UAS-crbintra (I-L) in conjunction with either UAS-yki RNAi (A, E, I), UAS-β-gal RNAi (B, F, J), UAS-kibra (C, G, K), or UAS-LacZ (D, H, L). Scale bars are 20μm. (M) Proportion of R8 cells that express Rh5 (magenta), Rh6 (green), or both (yellow). The error bars represent the standard deviation of total % Rh5 (% Rh5 + % Rh5+Rh6). Total % Rh5 was compared with two-sided, unpaired t-tests; * = p<0.01, ** = p<0.001, *** = p<0.0001. The shaded grey region between the dotted grey lines indicates wild type Rh5:Rh6 ratio range. lGMR>α-Spec RNAi>β-gal RNAi: n = 3 retinas, 342 ommatidia; lGMR>α-Spec RNAi>yki RNAi: n = 3, 725; lGMR>α-Spec RNAi>LacZ: n = 3, 436; lGMR>α-Spec RNAi>kibra: n = 3, 442; lGMR>kst RNAi>β-gal RNAi: n = 3, 462; lGMR>kst RNAi>yki RNAi: n = 4, 589; lGMR>kst RNAi>LacZ: n = 3, 908; lGMR>kst RNAi>kibra: n = 4, 791; lGMR>crbintra>β-gal RNAi: n = 3, 499; lGMR>crbintra>yki RNAi: n = 3, 835; lGMR>crbintra>LacZ: n = 3, 628; lGMR>crbintra>kibra: n = 3, 885. (TIF) [file pgen.1009146.s004.tif]

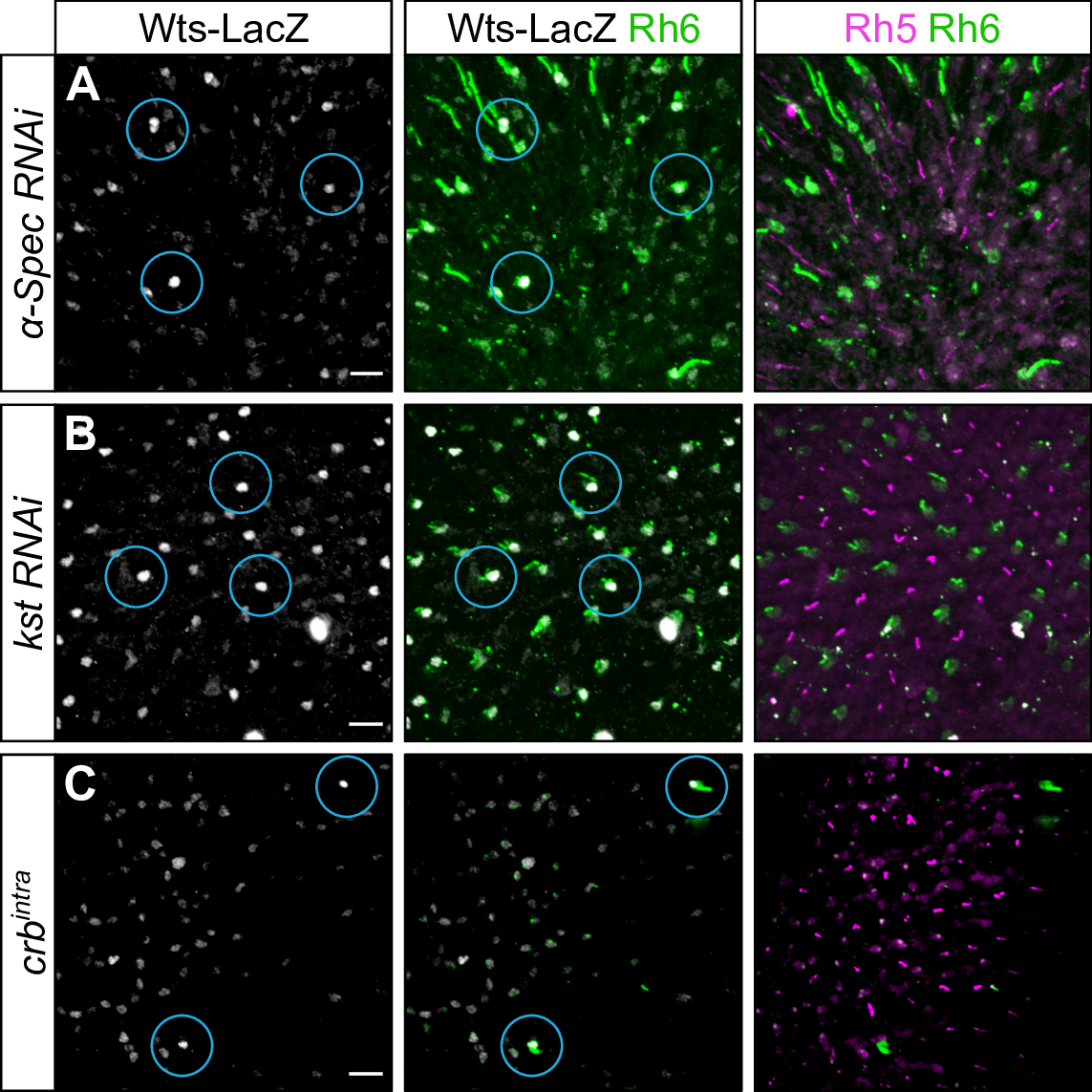

Supplement: S5 Fig — (A-C) Confocal microscope images of adult Drosophila retinas stained with anti-Rh5 (magenta), anti-Rh6 (green) and anti-β-gal (grey/orange; wts-LacZ) antibodies. Retinas expressed either UAS-α-Spec RNAi (A), UAS-kst RNAi (B), or UAS-crbintra (C). Blue circles indicate examples of wts-LacZ-positive cells which were also Rh6-positive. Scale bars are 20μm. Note that wts-LacZ is also expressed in the interommatidial cells, though at lower levels than in R8 cells; this is particularly evident in the retinas expressing crbintra. (TIF) [file pgen.1009146.s005.tif]

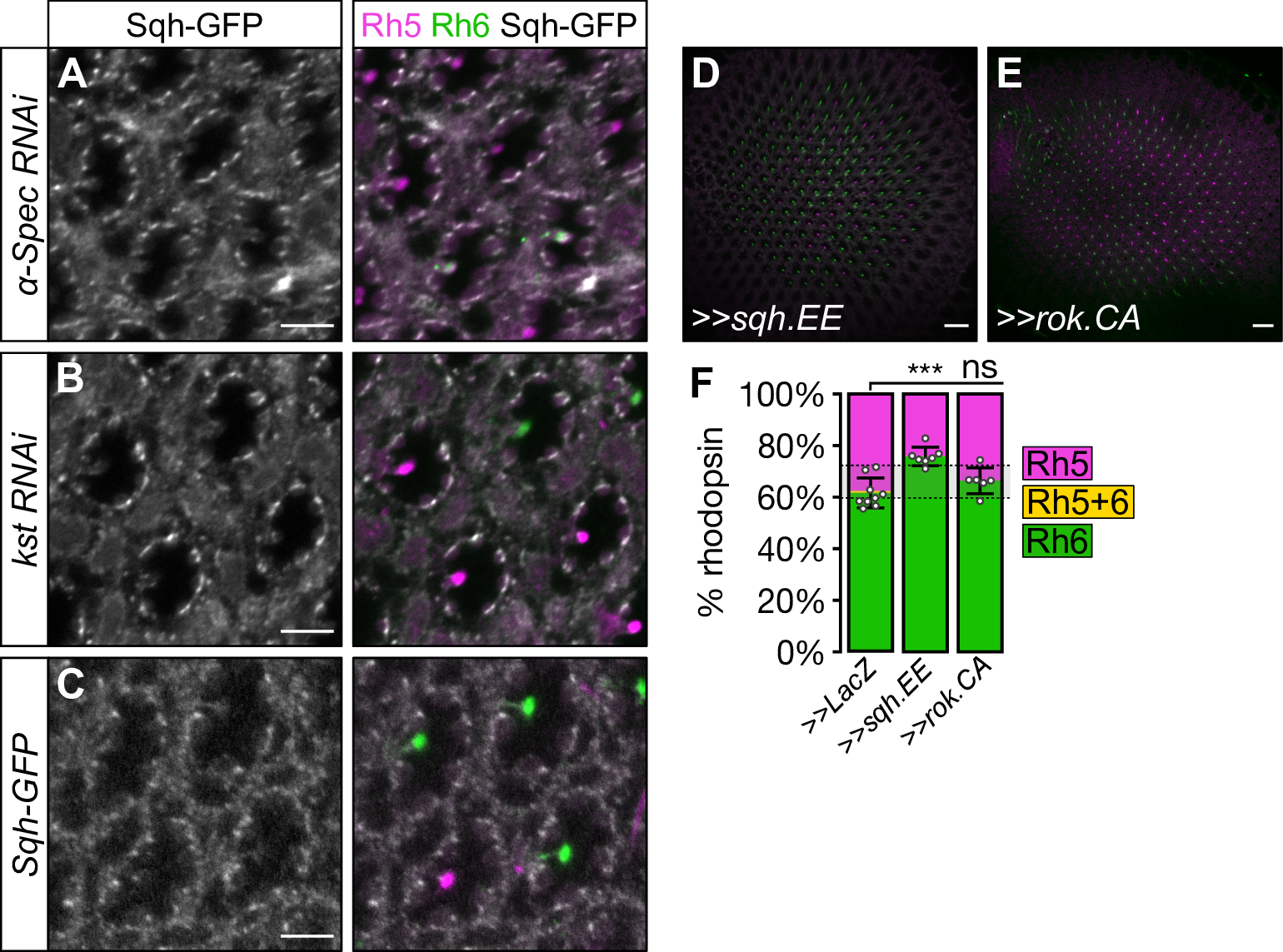

Supplement: S6 Fig — (A-C’) Confocal microscope images of adult Drosophila retinas stained with anti-Rh5 (magenta), and anti-Rh6 (green) antibodies showing Sqh-GFP localisation. Retinas expressed UAS-α-Spec RNAi (A), UAS-kst RNAi (B) or Sqh-GFP, alone (C). Scale bars are 10μm. (D-E) Confocal microscope images of adult Drosophila retinas stained with anti-Rh5 (magenta), and anti-Rh6 (green) antibodies. Retinas expressed UAS-sqh.EE (D), and UAS-rok.CA (E). Scale bars are 20μm. (F) Proportion of R8 cells that express Rh5 (magenta), Rh6 (green), or both (yellow). The error bars represent the standard deviation of total % Rh5 (% Rh5 + % Rh5+Rh6). Total % Rh5 was compared with two-sided, unpaired t-tests; ns = not significant, *** = p<0.0001. The shaded grey region between the dotted grey lines indicates wild type Rh5:Rh6 ratio range. >>LacZ: n = 9 retinas, 3211 ommatidia; >>sqh.EE: n = 7, 1355; >>rok.CA: n = 6, 1314. (TIF) [file pgen.1009146.s006.tif]

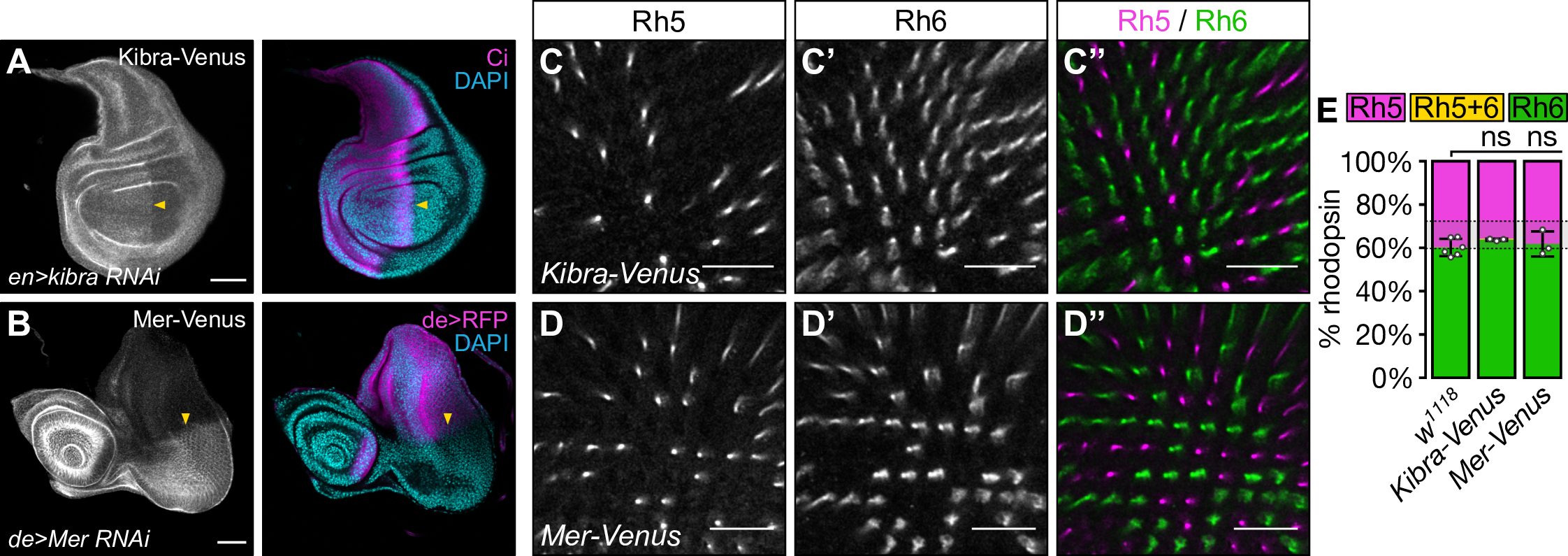

Supplement: S7 Fig — (A-B) Third instar larval wing (A) or eye (B) imaginal discs from D. melanogaster strains expressing endogenously tagged Kibra-Venus (A) or Mer-Venus (B). In (A), tissues were stained with anti-Ci antibody to mark the anterior (wild type) half of the wing disc, while in (B) RFP marks the dorsal (RNAi knockdown) half of the eye disc (magenta). DAPI (grey) marks nuclei. Genotypes are en-Gal4 / kibra RNAi; kibra-Venus / + (A) and Mer-Venus / +; de-Gal4, UAS-RFP / UAS-mer RNAi (B). In all images, anterior is towards the left and dorsal is towards the top. Scale bars are 100μm. Yellow arrowheads indicate a compartment boundary. (C-D”) Confocal microscope images of adult Drosophila retinas stained with anti-Rh5 (magenta) and anti-Rh6 (green) antibodies. Genotypes are kibra-Venus (C-C”) and mer-Venus (D-D”). Scale bars are 20μm. (E) Proportion of R8 cells that express Rh5 (magenta), Rh6 (green), or both (yellow). The error bars represent the standard deviation of total % Rh5 (% Rh5 + % Rh5+Rh6). Total % Rh5 was compared with two-sided, unpaired t-tests; ns = not significant. The shaded grey region between the dotted grey lines indicates wild type Rh5:Rh6 ratio range. kibra-Venus: n = 3 retinas, 864 ommatidia; mer-Venus: n = 3, 720. (TIF) [file pgen.1009146.s007.tif]
