# Supplementary material for: Crumbs and the apical spectrin cytoskeleton regulate R8 cell fate in the Drosophila eye
Source: PLoS Genet. 2021 Jun 7;17(6):e1009146. doi: 10.1371/journal.pgen.1009146 (PMC8211197; doi:10.1371/journal.pgen.1009146)
Supplement: Attachment — Submitted filename: Pojer Response to Reviewers.docx [file pgen.1009146.s008.docx]

We thank all three Reviewers and the Editor for their positive feedback and suggestions on how to improve our manuscript. We have discussed below how we have been able to respond to these suggestions. We apologise for the delays in revising the manuscript; the COVID-19 situation has had several significant time, personnel and financial impacts on our research.

We have added the following new data:

- **Figure 6 D-J** (examination in R8 cells of the influence of Crumbs on Kibra subcellular localisation and of Kibra on Merlin subcellular localisation)
- **Figure S2** (validation of Jub RNAi lines and Jub-GFP)
- **Figure S3 D-F** (examination of Yurt RNAi on R8 fate)
- **Figure S4** (Yki RNAi and Kibra overexpression epistasis experiments with modulation of Spectrins and Crumbs)
- **Figure S5** (*warts-lacZ* experiments with modulation of Spectrins and Crumbs)
- **Figure S6** (further experiments on Spaghetti Squash and R8 fate)
- **Figure S7** (validation of Kibra-venus and Merlin-venus strains)

**Reviewer #1**

In the present manuscript, Pojer et al explore the role of several upstream Hippo pathway components in R8 photoreceptor specification in the Drosophila retina. They find that, contrary to expectation based on past results in imaginal disc growth, the apical polarity determinants Crb and components of the Spectrin cytoskeleton function in an antagonistic manner in R8 specification. Furthermore, although apical Spectrin depletion leads to elevated pMyoII levels, the effects on R8 specification do not appear to be mediated bu Ajuba as in wing discs. Finally, Crb appears to promote the Yki-dependent “pale” R8 fate, possibly via an unknown FERM domain protein. The manuscript provided new insights into R8 fate specification and illustrate the fact that Hippo pathway upstream wiring is highly context-dependent. The manuscript should be of interest to the field, however, there are several aspects that can easily be improved. I would therefore support publication in PLoS Genetics, provided the points below are addressed.

1. The main shortcoming of the ms is that the Crb and Spectrin manipulations are not directly linked to Yki activity. This can be addressed as follows:
- Look at melted-LacZ and wts-LacZ expression upon loss of spectrin and crb, and upon overexpression of Crb-intra.
- Deplete yki in the context of Crb-intra overexpression and spectrin depletion, and measure R8 Rhosopsin expression.

We argue that data presented in the original manuscript do indeed show that Crb and Spectrin function are linked to Yki activity in R8 cells fate specification given that: a) Yki is a central mediator of R8 cell fate [1]; and b) in R8 cells *Rh5* is a direct target gene of Yki and its DNA-binding partner Sd [1], and we found that modulation of Crb or the apical spectrin cytoskeleton altered the prevalence of the pR8 subtype (which expresses the Yki target *Rh5*).

Nevertheless, to explore this further we performed the experiments suggested by the Reviewer. We depleted *yki* together with *α-Spec* or *kst*, or depleted *yki* and expressed *crb[intra]* and found that *yki* depletion was epistatic in each scenario (i.e. almost all R8 cells adopted the yR8 fate – **please see new Figure S4**)**.** We also assessed *wts-LacZ* expression upon depletion of spectrins or expression of crb-intra, given that *wts* is another target of Yki and Sd in R8 cells [1]. Consistent with our original conclusions, in R8 cells *wts-LacZ* was exclusively expressed in the **y**R8 subtype and its prevalence in R8 cells was modulated by *α-Spec RNAi*, *kst RNAi* and *crb[intra]* expression (**please see new Figure S5**)**.** Collectively, these results are consistent with the apical spectrin cytoskeleton and Crb modulating R8 cell fate by influencing Yki activity.

2. The authors show that pMyoII levels are elevated upon apical Spectrin depletion, yet Ajuba doesn’t appear to be involved as in wing discs. They should explore this further:
- Are the Ajuba RNAi lines they are using really working? Either show this by depletion in the wing disc or make Ajuba mutant clones in the retina.
- Does SqhEE overexpression affect R8 fate, which would suggest that a Jub-independent but MyoII-dependent mechanism downstream of Spectrin is at play here.

We could not assess a role for Jub in R8 cells using loss of function clones, as these grow poorly, owing to reduced Yki activity [2]. We assessed the functionality of the *jub RNAi* lines by expressing them in the posterior compartment of larval imaginal wing discs in a Jub-GFP background. We observed a strong reduction in Jub-GFP, which validates that these RNAi lines effectively deplete Jub (**please see new Figure S2**).

We misexpressed *sqh.EE* and *rok.CA* (constitutively active Rok, which phosphorylates Sqh) and observed a modest increase in the proportion of **y**R8 cells with *sqh.EE* overexpression (this is the opposite to what we would expect if *sqh.EE* promoted Yki activity in R8 cells, as has been reported in larval wing imaginal discs [3]) and no change with *rok.CA* overexpression (**please see** **new Figure S6**). This provides further validation of our original hypothesis that modulation of the apical spectrin cytoskeleton influences R8 cell fate independent of Sqh.

3. To try and identify the putative FERM domain protein involved in R8 specification together with Crb, the authors should try to deplete the main candidates Moesin and Yurt and look at Rhodopsin expression.

Yrt is a negative regulator of Crb during photoreceptor morphogenesis and interacts with the Crb FBM [4]. We depleted *yrt* in photoreceptor cells and found an increase in the proportion of **p**R8, which would be expected as Yrt was previously shown to be a negative regulator of Crb [4]. The mechanism by which Yrt modulates R8 fate choice requires further study, but this is beyond the scope of this manuscript. We did not assess the role of Moesin in R8 fate choice as Moesin has been shown to function as part of a complex with both Crb and Kst, interacting with and linking both proteins [5], so would be unlikely to regulate R8 cell fate with Crb and Kst, as we have found that these proteins have seemingly opposite influences on R8 cell fate choice.

4. Is Spectrin localisation changed in Crb-mutant clones? It would be interesting to compare the null with the delta-FBM allele.

Previously published work has shown that Kst is mislocalised to the lateral membrane in *crb* null mutant photoreceptor cells during the late pupal stage [6]. Our data show that the apical spectrin cytoskeleton and Crb have opposite roles in regulating R8 cell fate (**Fig. 2, 5**), suggesting that Crb and the apical spectrin cytoskeleton have been decoupled in regulating R8 cell fate. The mislocalistion of Kst upon Crb mutation cannot explain the results generated in this study.

Minor points:

1. Line 137: The authors state: “Depletion of α-Spec (40-60% pR8 cells across two RNAi lines, p=0.026,<0.0001)” but in Figure 2 one of the α-Spec RNAi lines is marked as not significant.

Thank you – this has been changed to read “(approximately 60% **p**R8 cells, p<0.0001; approximately 40% **p**R8 cells, p=ns)”. A significance cut off of 0.01 was used in this study.

2. Figure 4A-C: it looks like pSqh staining is reduced in IOCs upon Spectrin depletion. The authors should confirm whether this is the case and comment on this in the text.

pSqh is higher in IOCs than in photoreceptors in all three cases (**Fig. 4A-D**). However, as pSqh in photoreceptors is higher upon Spec depletion compared to control tissues, we think that levels in IOCs are similar across the different samples (**compare Fig. 4B/C with 4A**).

3. The Kibra-Venus/Mer-Venus transgene should be described in this manuscript.

The following sentences have been amended to describe the endogenously tagged Kibra-Venus and Merlin-Venus *Drosophila* strains that we made:

- “To do this, we generated an endogenously tagged Kibra-Venus D. melanogaster strain using CRISPR-Cas9 genome editing, which displayed a normal R8 subtype ratio indicating that Kibra function was not compromised (**please see new** **Figure S7A, C, E**).”
- “To visualise Mer in R8 cells, we generated an endogenously tagged Mer-Venus D. melanogaster strain using CRISPR-Cas9 genome editing, which displayed a normal R8 subtype ratio indicating that Mer function was not compromised (**please see new** **Figure S7B, D, E**).”

We have also presented experiments validating these endogenously tagged strains by RNAi **(please see new** **Figure S7):**

- Depletion of Kibra or Merlin by RNAi and visualization of the Venus-tagged proteins in larval imaginal discs (**Figure S7A-B**).
- Quantification of R8 subtypes in adult eyes from these strains which shows that there is no obvious perturbation of Kibra or Merlin function despite the addition of the Venus tag (**Figure S7C-E**).

The generation of these strains is also now described in the Methods section.

**Reviewer #2**

This is an interesting paper that sets out to study the relationship between the apical protein Crb, some of the proteins with which it has been associated, and the Hippo pathway in cell fate decision, using the fly eye as a model system.

The eye consists of 2 predominant subtypes of ommatidia. Approximately 30% express Rh3 in R7p and Rh5 in the paired R8p, while 70% express Rh4 in R7y and Rh6 in the paired R8y. Choice in Rhodopsin expression is stochastic and relies on a bistable feedback loop that involves components of the Hippo pathway. R8y fate is achieved through activation of Warts, which represses Yorkie activity. Inactivation of Wts promotes the R8p fate.
Crumbs is a known regulator of the Hippo pathway in growth control, but whether and how it might regulate cell fate decision in a cell type like the R8 photoreceptor, has not been explored in detail.

Overall, the paper is not easy to read, and this is largely due to the fact it presents many observations that are not always well integrated in a working model. While this could be addressed by working on the text, coming up with a clearer account of what might be going on will also require more experiments.

We have worked on the text to attempt to clarify it. Any blocks of new text that have been inserted into the revised manuscript have been highlighted in yellow to assist in the review process.

Panels appeared to be missing from Figure 6.

We are unclear on what the Reviewer is referring to regarding Figure 6. All panels are present, referenced in the main text and figure legend. For the different crb and kibra mutants (shown in panels A-B and quantified in C), examples of raw data for crb[11A22], crb[ΔFBM], and FRT82B are shown in **Figure 5A-C** and kibra[4] clones are shown in **Figure S2**.

The key findings reported here are that:
i) Crb is required during R8p / R8y cell fate decision. Loss of crb function leads to a reduction in R8p cells and increase in R8y. Conversely, overexpression of the Crb intracellular domain, which is known to harbour Crb signalling activity with respect to the Hippo pathway, greatly promotes the R8p fate. This is new and very interesting as it connects a known epithelial polarity determinant to cell fate decision.
ii) The Hippo pathway activator Kibra is a good candidate effector for Crb function in R8 fate decision. It has been proposed that Crb can inhibit the Hippo pathway by sequestering Kibra. Therefore, one might expect that loss of Crb would lead to activation of the Hippo pathway via kibra release. Consistent with this model, Kibra loss of function promotes the R8p fate.
iii) The spectrin cytoskeleton, and in particular the apical portion of it, which is known to interact with Crb, is part of the machinery that regulates R8 cell fate decision.

Collectively, these finding clearly point to a function for Crb and known interactors in R8 cell fate decision. However, the apparent contradiction between the Crb and Spectrin phenotypes and relative lack of a clear understanding of how exactly Kibra fits into this picture, makes it difficult go beyond the notion that things are complex and not do not behave as expected. So while the paper contains new interesting observations, collectively, no clear model comes out of the work. This could probably be addressed by further probing the links between Crb and Kibra and try for example to rescue the Crb loss of function by manipulating Kibra? Asking whether the effect of Crb overexpression on R8 fate decision dependents on Kibra? Similar experiments could be envisaged with respect to Spectrins. This would help to flesh out the functional connection between Kibra, Spectrin and Crb in R8.

We agree that Hippo signalling is very complex, especially at the level of the many different upstream inputs that feed into this pathway and cross-talk with each other in different contexts. In response to the suggestion by the Reviewer we point to data presented in the original manuscript and have also performed some new experiments

In the original manuscript, we assessed double *crb, kibra* double mutant tissue (with two *crb* alleles - *crb11A22* and *crbΔFBM* – **please see Figures 6A-C and S3C**), which addresses the first experiment suggested by the Reviewer. This data shows that Kibra loss is epistatic to Crb loss and suggests that Kibra acts downstream of, or in parallel to, Crb in the context of R8 cell fate choice.

We also performed additional experiments to probe the relationship between Crb, Kibra and Spectrins (**please see new Figure S4**). Here, we expressed *kibra* together with *crb-intra* or RNAi lines for different spectrins. In each case, *kibra* overexpression reverted the phenotype caused by *crb-intra* expression or apical *spectrins* depletion (**please see new Figure S4**). This reinforces our original conclusion that Kibra acts downstream of, or in parallel to both Crb and the apical spectrin cytoskeleton cells in the context of R8 cell fate choice.

I also think that the section related to P-Sqh might be problematic. P-Sqh antibodies are notoriously tricky to use and antibody penetration in the retina is not always good. MyoII has been shown to accumulate at the apical membrane in photoreceptors. The staining presented in Fig4 shows signal in the interommatidial cells, which becomes more diffused in kst and a-spec RNAI retina. No info is provided as to how, the intensity signal was quantified especially in light of the statement that ‘the apical spectrin cytoskeleton regulates phosphorylation of Sqh in R8 cell’– pp8.
I think that perhaps using the SqhGFP (or ZipGFP) fly strain might be more suitable, as it would bypass any technical limitations associated with the P-Sqh Ab. These GFP lines are very good proxies of “active MyoII”.

While antibody penetration into the retina can be bad, here, we have dissected out the eye and removed the lamina, so the R8 cell is at the most exposed part of the retina and these images are taken close to the base of the R8 cell to ensure highest antibody penetration.

Previous studies found that while depletion of the spectrin cytoskeleton increases levels of pSqh, it does not change overall Sqh levels [7]. To confirm this, we depleted *α-Spec* and *kst* in retinas also expressing Sqh-GFP and found no changes in Sqh-GFP levels (**please see new Figure S6A-C**).

The intensity signal for pSqh was quantified using Fiji/ImageJ. The following lines have been added to the Methods section (under the heading ‘**Image analysis and statistics**’):

- “To calculate pSqh antibody staining intensity, figures were cropped so only ommatidia with R8 cells were visible. The mean grey area was calculated from each figure using the Measurement tool.”

- The authors should discuss the possibility that by affecting photoreceptor morphogenesis, the crb lof might lead to failure in R7-R8 communication. This might well explain a requirement for the PDM domain.

We think that altered photoreceptor morphogenesis and a potential effect on R7-R8 communication are unlikely to underpin the *crb* mutant phenotype, for two reasons:

1) we observed the same increase in yR8 cells with the *crb* null allele (*crb[11A22]*), which also affects photoreceptor cell morphology [6], as we did with the *crbΔFBM* allele (**Figure 5A-D**), which harbours three point mutations in the FERM-binding domain and perturbs Crb signalling to the Hippo pathway but not the cell polarity and morphology-related functions of Crb [8–10].
2) the subcellular localisation of Crb and orientation of R7 and R8 cells in the optical column (**please see Figure 7 for details**), make it unlikely that extensive Crb-Crb extracellular domain binding occurs between R7 and R8 cells, which contrasts with the Crb-Crb binding that occurs between juxtaposed epithelial cells in larval imaginal discs.

We discussed both of these points in the original manuscript, but have added the following paragraph to the discussion to cover the former point in more detail:

- “Crb is known to play an important role in photoreceptor morphogenesis during pupal development [11]. Our results suggest that this role of Crb in regulating morphogenesis is distinct from its role in regulating R8 cell fate described here. We showed that mutating only the Crb FBM is sufficient to alter the R8 subtype ratio and this phenotype is present even in the absence of any defects in photoreceptor morphogenesis. We hypothesise that the role of Crb in R8 cell fate is also distinct from its role in regulating cell polarity, as mutating its PBM, which mediates the formation of the Crb polarity complex [12], failed to fully rescue the increased proportion of yR8 cells seen upon misexpression of *crb*.”

- Evaluating properly the work on Kibra and Mer requires one to have access to how the Venus transgenes were made. ‘To be described elsewhere’ prevents proper evaluation of this part of the work and is problematic. Are they functional transgenes? do they come with a phenotype? are they expressed at levels that are comparable to the endogenous, none tagged proteins, etc.

We have added details on the generation of the endogenously tagged Kibra-Venus and Merlin-Venus *Drosophila* strains that we made to the Methods. We have also provided evidence that these tagged proteins are functional as they do not affect R8 cell fate and can be depleted by RNAi (as described in comments to Reviewer 1 - **please also see new Figure S7**).

- It would be good to have an idea of how reliable the RNAi lines that are used in this study are, especially when the results are negative.

All of the Hippo pathway RNAi lines used in this study are effective at depleting their targets as they have been used in published studies and in some cases were used to identify roles for their target proteins in the Hippo pathway and organ growth control, e.g.:

| Gene | Stock number | References |
| --- | --- | --- |
| Yorkie | KK – VDRC 104523 | [13,14] |
| Warts | KK – VDRC 106174 | [1,14,15] |
| α-Spectrin | GD – VDRC 25387 | [7,16,17] |
|  | TRiP – BDSC 56932 | [18] |
| β-Spectrin | GD – VDRC 42053 | [16] |
|  | GD – VDRC 42054 | [7] |
|  | TRiP – BDSC 38533 | [18] |
| Karst | GD – VDRC 37074 | [16] |
|  | TRiP – BDSC 33933 | [15] |
| Ajuba | GD – VDRC 38442 | [3,19] |
|  | KK – VDRC 101993 | [19] |

- Please note that the sub-apical region (stalk in the photoreceptor) is not usually referred to as a 'junction'.

Thank you for picking up this error - we have changed ‘sub-apical junction’ to ‘sub-apical region’.

**Reviewer #3**

In this manuscript, Pojer et al investigate the role of the Hippo pathway in the control of fate choice in the R8 photoreceptors. The authors implicate Crumbs (Crb) and apical spectrin cytoskeleton regulators α-Spectrin (α-Spec) and Karst (Kst) in R8 cell fate determination. α-Spec and Kst are necessary for the specification of Rh6-expressing yR8 cells and knockdown of either gene resulted in a greater proportion of Rh5-positive pR8 cells in the adult retina. α-Spec and Kst knockdown also reduced phosphorylated Spaghetti squash (Sqh), but this did not alter warts activity through Ajuba activation. Alternatively, Crb is required for pR8 cell specification and mutations in the Crb and the FERM-binding domain alone significantly increase the proportion of Rh6-positive yR8 cells. This activity is independent of its role as a Kibra inhibitor. The authors conclude that only a subset of Hippo pathway proteins regulate the R8 binary cell fate decision. This study illustrates how the Hippo pathway mediates signal transduction in distinct biological settings and therefore it makes an important contribution to the Hippo field. Manuscript is well written, and the data are convincing.

Comments:
- Crb and spectrins were identified in a genetic screen which the authors plan to describe elsewhere. I suggest including it here, at least, as a summary table that shows how many genes were tested and how many positives were isolated. However, I do not consider this absolutely necessary and leave it at their discretion.

We appreciate this suggestion but have decided not to include more details of this screen in this manuscript as it forms the basis of a separate manuscript that is under review elsewhere.

- The authors use, at least, two RNAi lines to confirm the phenotypes, which is appropriate. Is it possible to confirm the efficiency of depletion by IF or RT-PCR/western blot? This is particularly important to substantiate the authors’ conclusions based on the lack of the phenotype in RNAi experiments (line 140, for example).

We have addressed this point above (in response to a similar point by Reviewer #2).

- Line 230 “Crb promotes yR8 cellfate” should be changed to “Crb promotes pR8 cellfate”.

Thank you for picking up this error - this has been corrected.

**References**

1.	Jukam D, Xie B, Rister J, Terrell D, Charlton-Perkins MA, Pistillo D, et al. Opposite feedbacks in the Hippo pathway for growth control and neural fate. Science. 2013;342: 1238016. doi:10.1126/science.1238016

2.	Das Thakur M, Feng Y, Jagannathan R, Seppa MJ, Skeath JB, Longmore GD. Ajuba LIM Proteins Are Negative Regulators of the Hippo Signaling Pathway. Curr Biol. 2010;20: 657–662. doi:10.1016/j.cub.2010.02.035

3.	Rauskolb C, Sun S, Sun G, Pan Y, Irvine KD. Cytoskeletal tension inhibits Hippo signaling through an Ajuba-Warts complex. Cell. 2014;158: 143–156. doi:10.1016/j.cell.2014.05.035

4.	Laprise P, Beronja S, Silva-Gagliardi NF, Pellikka M, Jensen M, McGlade CJ, et al. The FERM Protein Yurt Is a Negative Regulatory Component of the Crumbs Complex that Controls Epithelial Polarity and Apical Membrane Size. Dev Cell. 2006;11: 363–374. doi:10.1016/j.devcel.2006.06.001

5.	Medina E, Williams J, Klipfell E, Zarnescu DC, Thomas GH, Bivic AL. Crumbs interacts with moesin and βHeavy-spectrin in the apical membrane skeleton of Drosophila. J Cell Biol. 2002;158: 941–951. doi:10.1083/jcb.200203080

6.	Pellikka M, Tanentzapf G, Pinto M, Smith C, McGlade CJ, Ready DF, et al. Crumbs, the Drosophila homologue of human CRB1/RP12, is essential for photoreceptor morphogenesis. Nature. 2002;416: 143–149.

7.	Deng H, Wang W, Yu J, Zheng Y, Qing Y, Pan D. Spectrin regulates Hippo signaling by modulating cortical actomyosin activity. eLife. 2015;4: 1–17. doi:10.7554/eLife.06567

8.	Chen C-L, Gajewski KM, Hamaratoglu F, Bossuyt W, Sansores-Garcia L, Tao C, et al. The apical-basal cell polarity determinant Crumbs regulates Hippo signaling in Drosophila. Proc Natl Acad Sci U S A. 2010;107: 15810–15815. doi:10.1073/pnas.1004060107

9.	Ling C, Zheng Y, Yin F, Yu J, Huang J, Hong Y, et al. The apical transmembrane protein Crumbs functions as a tumor suppressor that regulates Hippo signaling by binding to Expanded. Proc Natl Acad Sci U S A. 2010;107: 10532–10537. doi:1004279107 [pii]\r10.1073/pnas.1004279107

10.	Robinson BS, Huang J, Hong Y, Moberg KH. Crumbs Regulates Salvador/Warts/Hippo Signaling in Drosophila via the FERM-Domain Protein Expanded. Curr Biol. 2010;20: 582–590. doi:10.1016/j.cub.2010.03.019

11.	Pellikka M, Tanentzapf G, Pinto M, Smith C, McGlade CJ, Ready DF, et al. Crumbs, the Drosophila homologue of human CRB1/RP12, is essential for photoreceptor morphogenesis. Nature. 2002;416: 143–149.

12.	Bulgakova NA, Knust E. The Crumbs complex: from epithelial-cell polarity to retinal degeneration. J Cell Sci. 2009;122: 2587–2596. doi:10.1242/jcs.023648

13.	Dent LG, Poon CLC, Zhang X, Degoutin JL, Tipping M, Veraksa A, et al. The GTPase regulatory proteins pix and git control tissue growth via the hippo pathway. Curr Biol. 2015;25: 124–130. doi:10.1016/j.cub.2014.11.041

14.	Poon CLC, Mitchell KA, Kondo S, Cheng LY, Harvey KF. The Hippo Pathway Regulates Neuroblasts and Brain Size in Drosophila melanogaster. Curr Biol. 2016;26: 1034–1042. doi:10.1016/j.cub.2016.02.009

15.	Forest E, Logeay R, Géminard C, Kantar D, Frayssinoux F, Milhavet LH, et al. The apical scaffold big bang binds to spectrins and regulates the growth of Drosophila melanogaster wing discs. J Cell Biol. 2018;11: 1–20.

16.	Fletcher GC, Elbediwy A, Khanal I, Ribeiro PS, Tapon N, Thompson BJ. The Spectrin cytoskeleton regulates the Hippo signalling pathway. EMBO J. 2015;34: 940–954. doi:10.15252/embj.201489642

17.	Deng H, Yang L, Wen P, Lei H, Blount P, Pan D. Spectrin couples cell shape, cortical tension, and Hippo signaling in retinal epithelial morphogenesis. J Cell Biol. 2020;219.

18.	Wong KKL, Li W, An Y, Duan Y, Li Z, Kang Y, et al. β-Spectrin Regulates the Hippo Signaling Pathway and Modulates the Basal Actin Network. J Biol Chem. 2015;290: 6397–6407. doi:10.1074/jbc.M114.629493

19.	Rauskolb C, Pan G, Reddy BVVG, Oh H, Irvine KD. Zyxin Links Fat Signaling to the Hippo Pathway. Basler K, editor. PLoS Biol. 2011;9: e1000624. doi:10.1371/journal.pbio.1000624
